# A stitch in time saves nine: closing the hole after removal of the aortic root cannula

**DOI:** 10.1186/1749-8090-4-2

**Published:** 2009-01-05

**Authors:** Ernesto Tappainer

**Affiliations:** 1 Cardiac Surgery Unit, "Carlo Poma" Hospital, 46100 Mantua, Italy

## Abstract

**Background:**

On completion of the surgical procedure the hole in the ascending aorta has to be closed after withdrawal of the aortic root cannula. The aorta is usually pinched by a double transversal stitch or it is crumpled by a purse string suture. Nevertheless, hemostasis is difficult to obtain because closure is done under recovered pressure. Additional stitches buttressed with teflon-felt pledgets are often required. Unfortunately, sensitivity to bacterial implantation and the proximity to the sternotomy line could make the foreign material of the pledgets responsible for chronic infections and fistulas.

**Methods:**

Two simple square stitches orthogonal to each other could be a very useful suture combining simplicity with effectiveness. To do this, two 4-0 polypropylene half-threads are put obliquely through the full thickness of the aortic wall, to and fro with inverse obliquities. Each of them draws a cross inside the aortic wall and two sides of a square outside. As a result a little square is drawn by the threads around the hole.

**Results:**

For years we have never needed to reinforce the closure by supplemental stitches with hundreds of patients.

**Conclusion:**

This type of closure has some advantages. In contrast to common stitches the aortic wall is not bent, crumpled or deformed, bites pass all aortic layers and the crossing of the threads covers the hole from inside rather than outside. Moreover, each thread can be tied with half of the tension required by other sutures because the two stitches act together but in the opposite direction. Finally, the technique is speedy and it requires only two half-threads. Most importantly, there is no need for teflon-felt pledgets. As a result, we have no longer seen any type of chronic infection or fistula.

## Background

**Figure 1:**
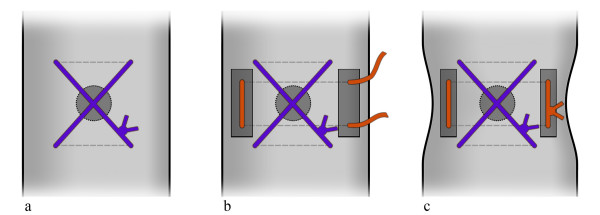
**Continous lines show threads on the external surface of the aorta**. Dotted lines show the internal passages of the needle as well the threads inside the aorta. a) The common single transversal stitch which is obtained from 2 passages of the needle. The passages are both in the same direction and orthogonal to the aorta. The X of the threads is outside and above the aortic hole. b) Reinforcement by a supplemental stitch with teflon-felt pledgets. The 2 passages of the needle are transversal again. c) The aorta is deformed after the stitches are tied.

After removal of the aortic root cannula (ARC), the closure of the site of insertion sometimes requires stitches reinforced with teflon-felt pledgets for haemostasis (Fig. 1). Unfortunately, sensitivity to bacterial implantation and the proximity to the sternotomy line could make the foreign material of the pledgets responsible for chronic infections and fistulas. With the aim of avoiding bleeding and particularly the use of prosthetic material like teflon-felt pledgets, we propose a different method of stitching the aortic hole.

## Methods

**Figure 2:**
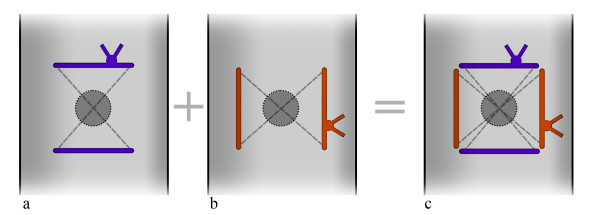
**Continous lines show threads on the external surface of the aorta**. Dotted lines show the internal passages of the needle as well the threads inside the aorta. a) In contrast to the previous technique the passages of the needle are oblique to the ascending aorta and made in the opposite direction. The X of the threads is inside the aorta and it covers the hole from the bottom. b) In the second stitch the 2 passages of the needle are the same as in the first but the whole second stitch acts in the opposite direction to the first. c) a square of the threads is completed outside the aorta around the hole. Each stitch is tied with half of the tension required in the previous technique. The aorta is not deformed. Supplemental stitches and teflon-felt pledgets are not necessary.

**Figure 3:**
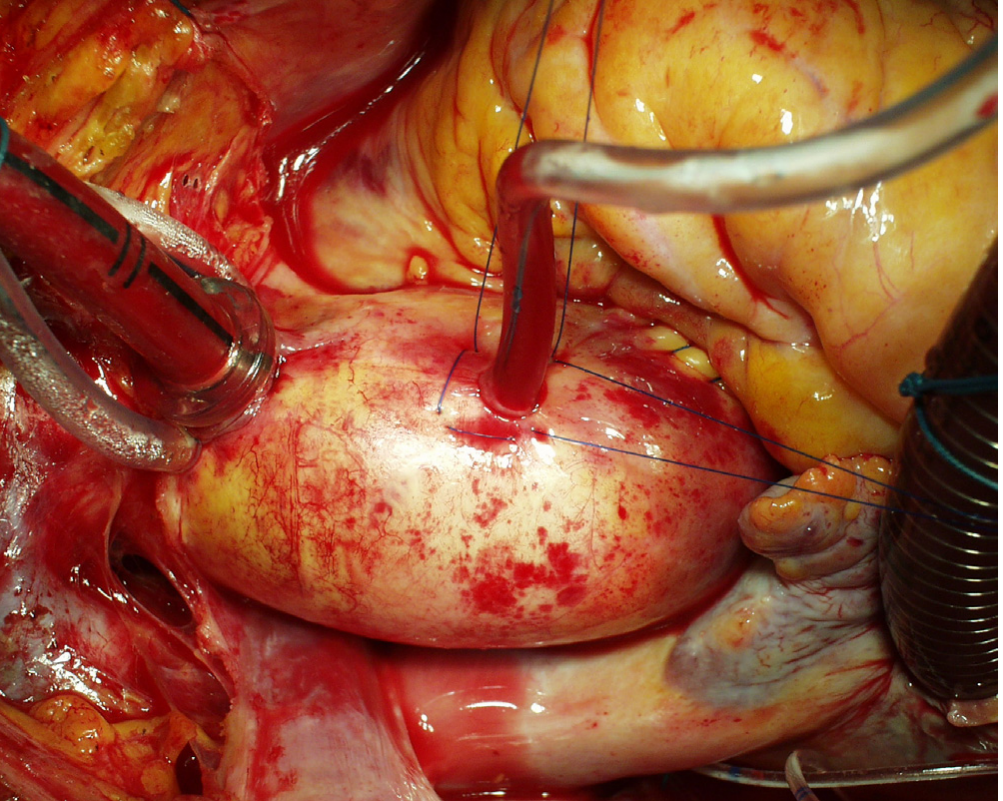
**The two orthogonal full-thickness stitches**. The blood is stopped at this moment by pulling the tourniquet which until now has fastened the aortic root cannula. The procedure, in this case, was an aortic valve replacement.

**Figure 4:**
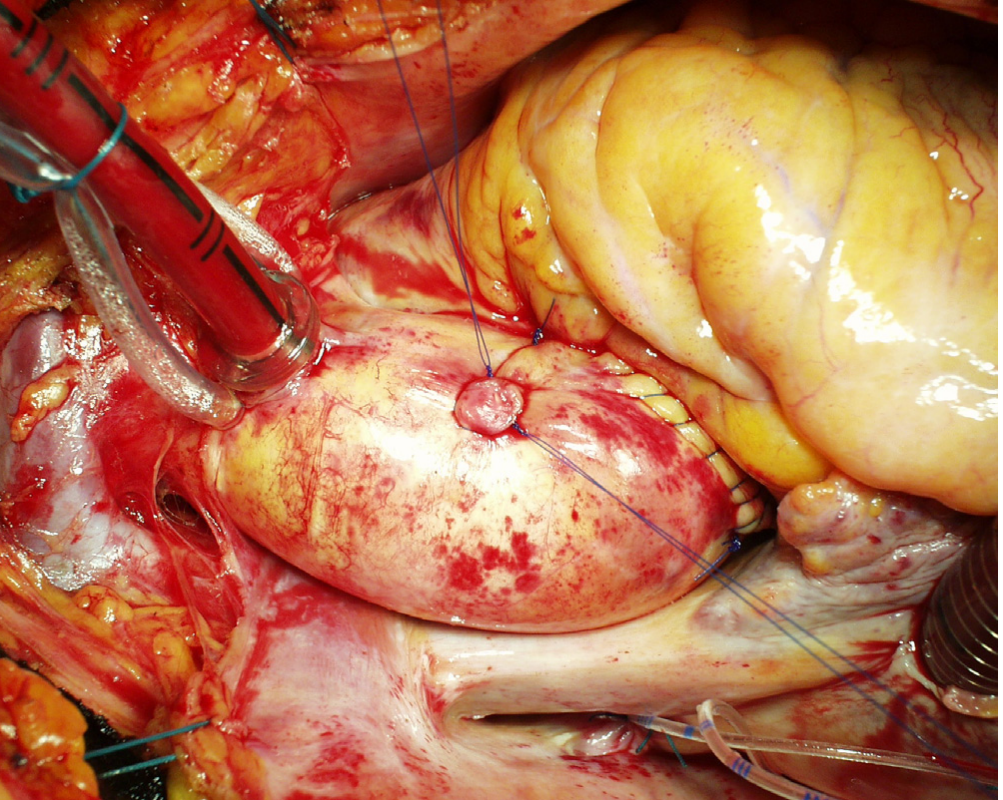
**The square drawn by the two stitches**. The adventitial thread in the tourniquet can be removed or tied.

We found that two simple square stitches orthogonal to each other (Fig. 2) could be a very useful suture combining simplicity with effectiveness. To do it, when the ARC is pulled out at the end of the main procedure, we plug the hole with a finger and pull the tourniquet to stop bleeding. Then, a 4-0 polypropylene thread, with half-circle needles, is divided into two halves. The first half-thread is put obliquely through the full thickness of the aortic wall to and fro with an inverse obliquity. In this way the thread draws a crossing (X) inside the aorta while it draws two sides of a square outside (Fig. 2a). The second half-thread does the same thing but in an orthogonal direction (Fig. 2b). It draws another X inside and completes the square outside, around the hole to be closed (Fig. 2c, Fig. 3, Fig. 4).

## Results

No comparison or study was planned for this elementary technique. We observed the positive results of the method over the years. In hundreds of patients there was no need to reinforce the closure by supplemental stitches with or without teflon-felt pledgets. Moreover, though sometimes wound dehiscence and infections obviously occurred even in our patients, none of them resulted in a remaining chronic infection or fistula.

## Discussion

Aortic root cannula (ARC) is a standard component of the cannulation set [1]. It is a small cannula, which is inserted by a big needle in the ascending aorta, proximal to the clamp site. ARC has many functions: delivering the antegrade cardioplegia, suction of the retrograde cardioplegic solution, aortic venting of the left ventricle and finally at the end of the surgical procedure it is useful for de-airing manoeuvres. On completion of the surgical procedure, to close the hole after withdrawal of the ARC, the aortic wall is usually pinched by transversal stitches (Fig. 1a) or alternatively it is crumpled by a purse-string suture. Nevertheless, hemostasis is sometimes difficult to obtain because closure is done under normal, recovered aortic pressure. Some problems could arise by the usual techniques such as bleeding, need for re-exploration, need for multiple sutures with prosthetic materials, but even dissection [2] or rupture [3] have been described. It often requires additional stitches buttressed with teflon-felt pledgets (Fig. 1b). For safety's sake, some surgeons use felt pledgets routinely.

Two aspects could be considered for the closure of this small hole. The first relates to the use of teflon-felt pledgets and the second to how the stitches work.

Prosthetic material, especially when porous, is highly sensitive to bacterial implantation and, in some patients, it is responsible for chronic infections and fistulas [4]. The usual site of ARC insertion – i.e. the anterior aspect of the ascending aorta – is very near to the posterior aspect of the sternal bone. After the thymus remnant is divided and the pericardial sac left open, the anterior aspect of the ascending aorta becomes very near to the posterior aspect of the sternal bone and the cut line, which is a frequent site of wound infection. Even better, transient contamination is always present during surgical procedures and such contamination is usually counteracted by the immunocompetent system of the patient [5]. Indeed, other factors favour bacterial implantation: the exposition of the sternotomy incision for a long time, the necrosis of the border of periostium after cauterization, the contact of the sternal wound with many foreign materials (bone wax, steel wires, subcutaneous threads, irritating disinfectant solutions, which could go down by capillarity). The combination of all these factors – i.e. the proximity of the porous material to the sternal incision and the exposure of the wound to transient contamination, foreign materials and traumatic manoeuvres – could lead to infection of the felt pledgets themselves. Occasionally, we found in redo procedures localized pus collection or cystic formation around teflon-felt pledgets and we noted that this occurred if they were near the sternotomy line. Moreover, in our country a chronic fistula, which at the end of repetitive treatments was found to be dependent on the felt pledgets, led to a court case. We think that the usual ARC site is much less protected than other sites which could require hemostatic reinforcement with teflon-felt pledgets. For example, the site of arterial cannulation is covered by the thymus remnant, the aortotomy in aortic valve replacement is covered by the right ventricle (Fig. 3). Indeed, ARC is usually placed on the uppermost point of the ascending aorta to facilitate de-airing. Unfortunately, this is the uncovered, most proximal point to the sternotomy line. In this way the unavoidable, temporary and minor bacterial contamination of the surgical wound may result in a definitive implantation in the porous material of the pledgets.

As regards how the stitches work, we noted that the common transversal closure by a single stitch – where the needle does two passages cranially and caudally to the hole (Fig. 1a) – pinches the aortic wall transversally often giving a sandglass aspect. Additional stitches buttressed with teflon pledgets, if needed for safety's sake, emphasize this non-natural appearance (Fig. 1c). Moreover, in the common closure by a single stitch the 2 transversal passages of the needle creates an X of the threads which lies over the hole on the external side of the aortic wall only (Fig. 1a). The corresponding hole in the intima remains uncovered and is not protected to prevent bleeding or dissection [2]. In our technique the passages of the needle are oblique rather than transversal (Fig. 2a, 2b). In this way the X is placed inside the aorta, thus covering the intimal hole from inside, while a small square is drawn around the hole outside (Fig. 2c, Fig. 3, Fig. 4).

In the case of the alternative purse-string suture, the aortic wall around the hole is crumpled by pulling a single thread. This creates many little pleats through which a dripping of blood could persist. This requires additional stitches, often with teflon-felt pledgets. Moreover, as the purse-string is as small as possible, the needle may not reach the intimal layer, which leaves a risk of dissection [2] or rupture [3] behind. Using our technique full thickness bites pass all layers of the aortic wall which is not bent or crumpled and the aorta is not deformed (Fig. 4). Moreover, each thread can be tied with half of the tension required by other techniques because the two stitches act together but in the opposite direction (Fig. 2c, Fig. 3).

## Conclusion

Our method offers some advantages. The aortic wall is not bent, crumpled or deformed as with common stitches. The crossing of the threads covers and closes the hole from inside rather than outside. Bites pass all aortic layers. The technique is also speedy and it requires only two half-threads. Each half-thread can be tied with half of the strain too. Finally, for years reinforcement of the closure was no longer needed with hundreds of patient and most significantly the use of teflon-felt pledgets was stopped. Accordingly, we have no longer seen any type of remaining chronic infection or fistula.

## Competing interests

The author declares that they have no competing interests.

## Authors' contributions

The technique here described has been conceived and adopted by the author
